# Solving Operating Room Scheduling Problem Using Artificial Bee Colony Algorithm

**DOI:** 10.3390/healthcare9020152

**Published:** 2021-02-02

**Authors:** Yang-Kuei Lin, Min-Yang Li

**Affiliations:** Department of Industrial Engineering and Systems Management, Feng Chia University, Taichung 407, Taiwan; minyli@fcu.edu.tw

**Keywords:** scheduling, operating rooms, artificial bee colony, heuristic

## Abstract

Many healthcare institutions are interested in reducing costs and in maintaining a good quality of care. The operating room department is typically one of the most costly units in a hospital. Hospital managers are always interested in finding effective ways of using operating rooms to minimize operating costs. In this research, we study the operating room scheduling problem. We consider the use of a weekly surgery schedule with an open scheduling strategy that takes into account the availabilities of surgeons and operating rooms. The objective is to minimize the total operating cost while maximizing the utilization of the operating rooms but also minimizing overtime use. A revised mathematical model is proposed that can provide optimal solutions for a surgery size up to 110 surgical cases. Next, two modified heuristics, based on the earliest due date (EDD) and longest processing time (LPT) rules, are proposed to quickly find feasible solutions to the studied problem. Finally, an artificial bee colony (ABC) algorithm that incorporates the initial solutions, a recovery scheme, local search schemes, and an elitism strategy is proposed. The computational results show that, for a surgery size between 40 and 100 surgical cases, the ABC algorithm found optimal solutions to all of the tested problems. For surgery sizes larger than 110 surgical cases, the ABC algorithm performed significantly better than the two proposed heuristics. The computational results indicate that the proposed ABC is promising and capable of solving large problems.

## 1. Introduction

In most hospitals, the operating room department is both a high-cost and a high-revenue unit. Hospital managers are always seeking methods to devise schedules that maximize the utilization of operating rooms but also minimize the total cost. An open scheduling strategy is applied in some hospitals, i.e., surgeons can choose any workday for their surgeries, and anesthetists and nurses cooperate with the surgeons to maximize efficiency. In this paper, we study an operating room scheduling problem. We consider the use of a weekly surgery schedule with an open scheduling strategy that takes into account the availabilities of surgeons and operating rooms. The objective is to minimize the total operating cost while maximizing the utilization of the operating rooms but also minimizing overtime use.

First, we assigned a set of surgeries Ω to $M_{d}$ operating rooms within the planning horizon (*H*) to minimize the total operating cost. Each surgery has an operating duration $t_{i}$ and a due date $D_{i}$. If the due date of a surgery falls within the current planning horizon ($D_{i}\leq H$), it has to be scheduled therein. However, if the due date is later than the planning horizon ($D_{i}>H$), it can either be scheduled in the current planning horizon or postponed to a later date. All surgeries have to be scheduled no later than their due dates ($d_{i}\leq D_{i}$). Each surgery can only be scheduled at most once over the planning horizon. Once a surgery starts, it has to continue until it is finished. Each surgery is assigned to a specific surgeon in advance. $\Omega_{l}$ indicates a set of surgeries that are assigned to surgeon *l*. Every surgeon has a maximum operating time for day *d* ($A_{l}^{d}$); overtime is not allowed. We assume that all operating rooms are multifunctional. Each operating room can be used for any of the scheduled surgeries. Anesthesia and nursing staff and equipment are always available when needed. Each operating room *k* on day *d* has regular opening hours ($RT_{k}^{d}$) and a maximum permissible overtime ($OT_{k}^{d}$). The hospital manager wants to maximize the utilization of operating rooms. If the total operating time scheduled for operating room *k* on day *t* ($f_{k}^{d})$ is less than its regular opening hours, waste cost $UC_{k}^{d}\;=\;\max\left(0,RT_{k}^{d}-f_{k}^{d}\right)\;\forall k,d$ is incorporated, thereby discouraging unused time. Overtime-operating cost $OC_{k}^{d}\;=\;\max\left(0,f_{k}^{d}-RT_{k}^{d}\right)\;\forall k,d$ is also introduced if the total operating time scheduled for operating room *k* on day *t* ($f_{k}^{d})$ exceeds its regular opening hours. We assume that the waste cost equals the amount of unused time while the overtime-operating cost equals the amount of overtime multiplied by a “penalty” value (α). The objective is to minimize the total operating cost, i.e., min $\sum_{d\;=\;1}^{H}\sum_{k\;=\;1}^{m_{d}}C_{k}^{d}$ where $C_{k}^{d}\;=\;UC_{k}^{d}\;+\;\alpha OC_{k}^{d}$.

Due to its importance in practice, more and more researchers have focused their attention on the operating room scheduling problem in recent years [1,2,3,4,5,6,7,8,9,10,11,12]. Some researchers have attempted to assign surgeries to operating rooms to maximize the efficiency of operating room utilization and to minimize idle time and overtime costs [1,2]. However, in practice, before surgery, patients are usually treated by a specific surgeon who performed the diagnosis and provided consultation. Hence, surgery is typically assigned to a specific surgeon. Whenever surgery is assigned to a specific day, the related surgical staff will be allocated by the operating planning manager.

In tackling this problem, some researchers have taken into account the availabilities of surgeons and operating rooms [3,4] while others have considered the availabilities of multiple resources (such as operating rooms, surgeons, equipment, and recovery beds) [5,6,7,8,9]. Recently, some researchers studied a daily surgery scheduling problem taking into account multiple resources and stages, i.e., pre-surgery, surgery, and post-surgery; such an approach in which the different resources required in each stage are taken into account is called “comprehensive operating room scheduling”. In the present research, the “no-wait” constraint is considered between the three stages. Surgeries are known in advance (i.e., were elective in nature). The allocation of resources is considered throughout the process: pre-operative holding units (PHU) beds in the first stage; operating rooms, surgeons, nurses, and anesthetists in the second stage; and post anesthesia care unit (PACU) beds in the third stage. The objective is to minimize the maximum end time of the last activity in stage 3 and the total idle time in the operating rooms [10,11,12]. As more resources and stages are considered, operating scheduling problems become progressively more complicated.

This research considers a similar problem to that addressed in [3,4]. In [3], the authors studied a tactical operating room planning problem by taking into account the availabilities of surgeons and operating rooms. The objectives were to maximize operating room utilization and to minimize overtime cost. They first constructed a mathematical model that assigned surgeries to operating rooms over a period of one week by using an open scheduling strategy. Next, they reformulated the mathematical model as a set-partitioning model and then solved it by using a column-generation-based, heuristic procedure with four criteria. In [4], the authors studied a dynamic scheduling problem that consisted of three stages: the first was to assign operating days to each specialty; the second was to address the surgeon assignment problem; and the third was to deal with the assignment and sequence of patients. The objective was to minimize total patient waiting and operating room overtime costs. Zhu et al. [4] used a hybrid Grey Wolf Optimizer)–Variable Neighborhood Search (GWO-VNS) algorithm combining the Grey Wolf Optimizer (GWO) with a Variable Neighborhood Search (VNS). The operating room scheduling problem was shown to be NP-hard through a reduction to the 0–1 multiple knapsack problem [13].

The operating room department is typically the unit with the highest costs and revenue. Hence, it is important to ensure the efficient use of operating rooms. The purpose of this research is to obtain an efficient operating schedule that maximizes the utilization of operating rooms while minimizing idle time and overtime. Considering the complexity of operating room scheduling problems, we propose the use of easy-to-implement heuristics and an artificial bee colony (ABC) to solve the studied problem efficiently.

## 2. Mathematical Model

We considered assigning a set of surgeries to operating rooms within a planning horizon to minimize total operating cost. The notations used in this section are given in Table 1.

The studied problem was formulated as a mathematical model by Fei et al. [3]. In [3], the objective function was formulated as Min $\sum_{d\;=\;1}^{N_{D}}\sum_{k\;=\;1}^{M_{d}}C_{k}^{d}\;=\;\max\left\{\left(R_{k}^{d}-\sum_{i\in \Omega }t_{i}z_{ik}^{d}\right),\beta \left(\sum_{i\in \Omega }t_{i}z_{ik}^{d}-R_{k}^{d}\right)\right\}$. The objective function is nonlinear due to the max term. Also, the work in [3] failed to consider Constraint (4). Without Constraint (4), a surgery *i* with $D_{i}\leq H$ might be scheduled in day *d* = 1 to *D_i_* first and then may be scheduled in day *d* = *D_i_* to *H* again. It violates the condition that every surgery can only be scheduled at most once over the planning horizon, and hence, it is infeasible. We modified the model in [3] so that it can find an optimal solution for the studied problem. First, we revised the objective function in [3] as Constraints (1), (7), and (8) so that the objective function is linear. Second, we added Constraint (4) to prevent a surgery from being scheduled twice. The modified mixed integer programming (MIP) model for the studied problem is as follows:

Decision variable $x_{ik}^{d}\;=\;1$ if surgery *i* is assigned to operating room *k* on day *d*; otherwise = 0. The objective function seeks to minimize the cost of total unused time and overtime. Constraint (2) denotes that, if a surgery’s deadline is smaller or equal to *H*, it should be scheduled exactly once before its deadline. Constraint (3) indicates that, if a surgery’s deadline is larger than *H*, it schedules at most once over the planning horizon. Constraint (4) ensures that each surgery can only be scheduled at most once over the planning horizon. Constraint (5) ensures that the total operating time of any operating room on any planning day would not exceed its maximum permissible overtime. Constraint (6) ensures that the total operating time assigned to each surgeon per day cannot exceed their maximum working hours on that day. Constraints (7) and (8) ensure that $C_{k}^{d}$ takes either the value of waste cost for the unused time of operating room *k* on day *d* or the value of α times the overtime operating cost of operating room *k* on day *d* depending on which value is larger. Constraints (9) and (10) show the nonnegativity and integrality restrictions.
$\mathrm{objective}:\;\mathrm{Min}\;\sum_{d\;=\;1}^{H}\sum_{k\;=\;1}^{M_{d}}C_{k}^{d}$(1)$\mathrm{subject}\;\mathrm{to}:\;\sum_{d\;=\;1}^{D_{i}}\sum_{k\;=\;1}^{M_{d}}x_{ik}^{d}\;=\;1\;\mathrm{for}\;i\in \Omega \;and\;D_{i}\leq H$(2)$\overset{H}{\underset{d\;=\;1}{\sum }}\overset{M_{d}}{\underset{k\;=\;1}{\sum }}x_{ik}^{d}\leq 1\;\mathrm{for}\;i\in \Omega \;and\;D_{i}>H$(3)$\overset{H}{\underset{d\;=\;1}{\sum }}\overset{M_{d}}{\underset{k\;=\;1}{\sum }}x_{ik}^{d}\leq 1\;\mathrm{for}\;i\in \Omega$(4)$\underset{i\in \Omega }{\sum }t_{i}x_{ik}^{d}\leq RT_{k}^{d}\;+\;OT_{k}^{d}\;\mathrm{for}\;k\;=\;1,\ldots ,M_{d};d\;=\;1,\;\ldots ,\;H$(5)$\overset{M_{d}}{\underset{k\;=\;1}{\sum }}\overset{}{\underset{i\in \Omega_{l}}{\sum }}t_{i}x_{ik}^{d}\leq A_{l}^{d},d\;=\;1,\;\ldots ,\;H;l\;=\;1,\;\ldots ,\;L$(6)$C_{k}^{d}\geq RT_{k}^{d}-\sum_{i\in \Omega }t_{i}x_{ik}^{d}\;\mathrm{for}\;k\;=\;1,\ldots ,M_{d};\;d\;=\;1,\;\ldots ,\;H$(7)$C_{k}^{d}\geq \alpha \left(\sum_{i\in \Omega }t_{i}x_{ik}^{d}-RT_{k}^{d}\right)\;\mathrm{for}\;k\;=\;1,\ldots ,M_{d};\;d\;=\;1,\;\ldots ,\;H$(8)$C_{k}^{d}\geq 0\;\mathrm{for}\;k\;=\;1,\ldots ,M_{d};\;d\;=\;1,\;\ldots ,\;H$(9)$x_{ik}^{d}\;=\;0\;\mathrm{or}\;1\;\mathrm{for}\;i\in \Omega ;k\;=\;1,\ldots ,M_{d};d\;=\;1,\;\ldots ,\;H$(10)

## 3. Heuristics

Two easy-to-implement heuristics based on the varied earliest due date (EDD) rule and varied longest processing time (LPT) rule are proposed for the studied problem. We describe the steps of the two heuristics as follows.

### 3.1. Modified EDD Heuristic (MEDD)

**Figure 1:**
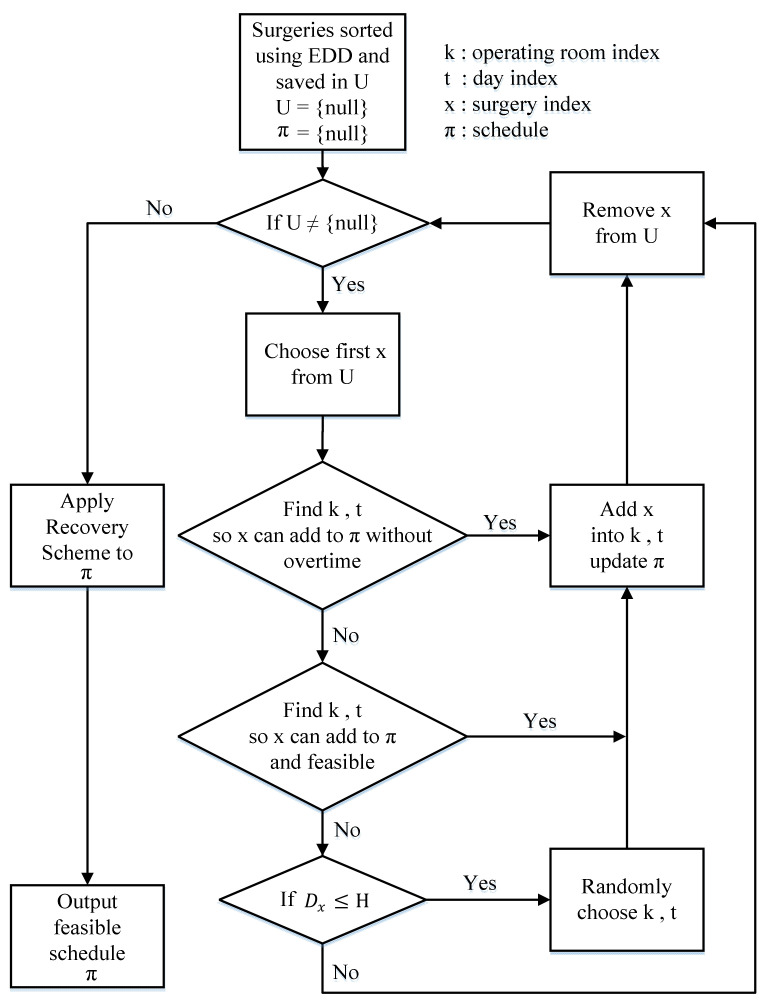
The flow chart of the modified earliest due date (MEDD) heuristic.

In step 1, the MEDD heuristic first sorts all surgeries in EDD order. It breaks ties by using the LPT rule. In step 2, the MEDD heuristic tries to find operating room *k* on day *d* that surgery *x* can be assigned to without exceeding the regular opening time of the operating room *k* and the maximum operating time available of surgeon $l,\;x\in \Omega_{l}$. The search starts from the first operating room *k* = 1 on first day *d* = 1 and ends on the last operating room *k* = $M_{d}$ on last day *d* = $D_{x}$. If *k* and *d* are found, then add surgery *x* into operating room *k* on day *d*, update schedule π, remove surgery *x* from U, and go back to step 2. In step 3, if surgery *x* cannot be assigned to solution π without exceeding the regular opening time of the operating room and the maximum operating time available of surgeon $l,\;x\in \Omega_{l}$. Then, the MEDD heuristic tries to find operating room *k* on day *d* that surgery *x* can be assigned to without exceeding the maximal permissible overtime of operating room and the maximum operating time available of surgeon $l,\;x\in \Omega_{l}$. If *k* and *d* are found, then add surgery *x* into operating room *k* on day *d*, update schedule π, remove surgery *x* from U, and go back to step 2. In step 4, if surgery *x* cannot be scheduled into schedule π after steps 2–3 and if $D_{x}\leq H$, then the MEDD heuristic randomly chooses one operating room *k* on day $d,\;d\leq D_{x}$. Add surgery x into operating room *k* on day *d*, update schedule π, remove surgery *x* from U, and go back to step 2. However, if $D_{x}>H$, then the MEDD heuristic will remove surgery x from U and go back to step 2. In the last step, step 5 applies RecoveryScheme to ensure that the generated schedule π is feasible. Figure 1 shows the flow chart of the MEDD heuristic.

#### MEDD Heuristic

Step 1. Sort all surgeries by the EDD rule. Break any ties using the LPT rule. Save all the sorted surgeries in set U. Initially, set schedule π = {null}.

Step 2. If $U\;=\;\left\{\mathrm{null}\right\}$, go to Step 5. Otherwise, try to schedule the first surgery x, x∈U, $x\in \Omega_{l}$ in operating room *k* on day *d* if it satisfies $f_{k}^{d}$($\pi )\leq RT_{k}^{d}$ and $g_{l}^{d}\left(\pi \right)\leq A_{l}^{d}$. Try *k* = 1 first; if *k* is not feasible, then try *k* = *k* + 1 until *k* >$\;M_{d}$. Similarly, try *d* = 1 first; if *d* = 1 is not feasible, then try *d* = *d* + 1 until *d* > $D_{x}$. If *k* and *d* are found, then add surgery *x* into operating room *k* on day *d*, update schedule π, remove surgery *x* from U, and go back to step 2.

Step 3. Try to schedule the first surgery $x,\;x\in U,\;x\in \Omega_{l}$ in operating room *k* on day *d* if it satisfies $f_{k}^{d}$($\pi )\leq RT_{k}^{d}\;+\;OT_{k}^{d}$ and $g_{l}^{d}\left(\pi \right)\leq A_{l}^{d}$. Try *k* = 1 first; if *k* is not feasible, then try *k* = *k* + 1 until k > $M_{d}$. Similarly, try *d* = 1 first; if *d* = 1 is not feasible, then try *d* = *d* + 1 until *d >* $D_{x}$. If *k* and *d* are found, then add surgery *x* into operating room *k* on day *d*, update schedule π, remove surgery *x* from U, and go back to step 2.

Step 4. For the first surgery x, x∈U, if $D_{x}\leq H$, then randomly choose one operating room *k* on day $d,\;d\leq D_{x}$. Add surgery x into operating room *k* on day *d*, update schedule π, remove surgery *x* from U, and go back to step 2. Otherwise, remove surgery *x* from U and go back to step 2.

Step 5. Apply RecoveryScheme (described in Section 4.3) to schedule π.

### 3.2. Modified LPT Heuristic (MLPT)

**Figure 2:**
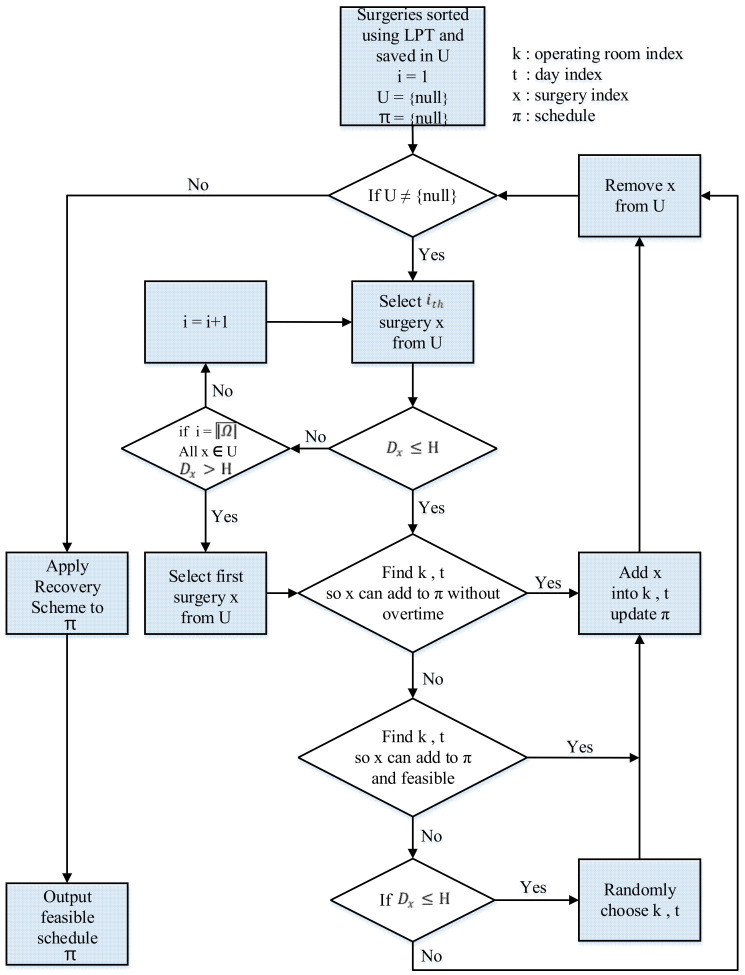
The flow chart of modified longest processing time (MLPT) heuristic.

In step 1, the MLPT heuristic first sorts all surgeries in LPT order. It breaks ties by using the EDD rule. Then, in steps 2–4, it handles surgeries that have due dates smaller than or equal to the planning horizon ($D_{x}\leq H$) based on their sorted order in U. In step 2, the MLPT heuristic tries to find operating room *k* on day *d* that surgery *x* can be assigned to without exceeding the regular opening time of the operating room *k* and the maximum operating time available of surgeon $l,\;x\in \Omega_{l}$. The search starts from the first operating room *k* = 1 on the first day *d* = 1 and ends at the last operating room *k* = $M_{d}$ on the last day *d* = $D_{x}$. If *k* and *d* are found, then add surgery *x* into operating room *k* on day *d*, update schedule π, remove surgery *x* from U, and go back to step 2. In step 3, if surgery *x* cannot be assigned into solution π without exceeding the regular opening time of the operating room and the maximum operating time available of surgeon $l,\;x\in \Omega_{l}$, then the MLPT heuristic tries to find operating room *k* on day *d* that surgery *x* can be assigned to without exceeding the maximal permissible overtime of operating room and the maximum operating time available of surgeon $l,\;x\in \Omega_{l}$. If *k* and *d* are found, then add surgery *x* into operating room *k* on day *d*, update schedule π, remove surgery *x* from U, and go back to step 2. In step 4, if surgery *x* cannot be scheduled into schedule π after steps 2–3, then the MLPT heuristic randomly chooses one operating room *k* on day $d,\;d\leq D_{x}$. Add surgery *x* into operating room *k* on day *d*, update schedule π, remove surgery *x* from U, and go back to step 2. steps 5–6, the MLPT heuristic handles surgeries that have due dates larger than the planning horizon ($D_{x}>H$). The procedures are similar to steps 2–3. If *k* and *d* are not found in steps 5–6, the MLPT does not consider scheduling *x* into schedule π anymore. It removes surgery *x* from U and goes back to step 5. In the last step, step 7 applies RecoveryScheme to ensure that the generated schedule π is feasible. Figure 2 shows the flow chart of the MLPT heuristic.

#### MLPT Heuristic

Step 1. Sort all surgeries by the LPT rule. Break any ties by using the EDD rule. Save all the sorted surgeries in set U. Initially, set schedule π = {null}.

Step 2. If $U\;=\;\left\{\mathrm{null}\right\}$, go to step 7. Otherwise, selects the first surgery x, $x\in U,\;x\in \Omega_{l}$ that satisfies $D_{x}\leq H$. Try to schedule surgery x in operating room *k* on day *d* if it satisfies $f_{k}^{d}$($\pi )\leq RT_{k}^{d}$ and $g_{l}^{d}\left(\pi \right)\leq A_{l}^{d}$. Try *k* = 1 first; if *k* is not feasible, then try *k* = *k* + 1 until k >$\;M_{d}$. Similarly, try *d* = 1 first; if *d* = 1 is not feasible, then try *d* = *d* + 1 until *d >*$\;D_{x}$. If *k* and *d* are found, then add surgery *x* into operating room *k* on day *d*, update schedule π, remove surgery *x* from U, and go back to step 2.

Step 3. Select the first surgery x, $x\in U,\;x\in \Omega_{l}$ that satisfies $D_{x}\leq H$. Try to schedule surgery x into operating room *k* on day *d* if it satisfies $f_{k}^{d}$($\pi )\leq RT_{k}^{d}\;+\;OT_{k}^{d}$ and $g_{l}^{d}\left(\pi \right)\leq A_{l}^{d}$. Try *k* = 1 first; if *k* is not feasible, then try *k* = *k* + 1 until *k* > $M_{d}$. Similarly, try *d* = 1 first; if *d* = 1 is not feasible, then try *d* = *d* + 1 until *d >*$\;D_{x}$. If *k* and *d* are found, then add surgery *x* into operating room *k* on day *d*, update schedule π, remove surgery *x* from U, and go back to step 2.

Step 4. Select the first surgery x, $x\in U,\;x\in \Omega_{l}$ that satisfies $D_{x}\leq H$. Randomly choose one operating room *k* on day $d,\;d\leq D_{x}$. Add surgery *x* into operating room *k* on day *d*, update schedule π, remove surgery *x* from U, and go back to step 2.

Step 5. If $U\;=\;\left\{\mathrm{null}\right\}$, go to step 7. Otherwise, select the first surgery x, $x\in U,\;x\in \Omega_{l}$. Try to schedule surgery x into operating room *k* on day *d* if it satisfies $f_{k}^{d}$($\pi )\leq RT_{k}^{d}$ and $g_{l}^{d}\left(\pi \right)\leq A_{l}^{d}$. Try *k* = 1 first; if *k* is not feasible, then try *k* = *k* + 1 until k > $M_{d}$. Similarly, try *d* = 1 first; if *d* = 1 is not feasible, then try *d* = *d* + 1 until *d >* H. If *k* and *d* are found, then add surgery *x* into operating room *k* on day *d*, update schedule π, remove surgery *x* from U, and go back to step 5.

Step 6. Select the first surgery x, $x\in U,\;x\in \Omega_{l}$. Try to schedule surgery x into operating room *k* on day *d* if it satisfies $f_{k}^{d}$($\pi )\leq RT_{k}^{d}\;+\;OT_{k}^{d}$ and $g_{l}^{d}\left(\pi \right)\leq A_{l}^{d}$. Try *k* = 1 first; if *k* is not feasible, then try *k* = *k* + 1 until k >$\;M_{d}$. Similarly, try *d* = 1 first; if *d* = 1 is not feasible, then try *d* = *d* + 1 until *d >* H. If *k* and *d* are found, then add surgery *x* into operating room *k* on day *d*, update schedule π, remove surgery *x* from U, and go back to step 5. If *k* and *d* are not found, then remove surgery *x* from U and go back to step 5.

Step 7. Apply RecoveryScheme (described in Section 4.3) to schedule π.

## 4. Artificial Bee Colony (ABC) Algorithm

The ABC algorithm was originally proposed by Karaboga [14]. It is a newly developed metaheuristic that simulates bees’ food search behavior. Compared to other metaheuristics, it has the advantages of being simple, flexible, and robust [15,16]. According to [1], the surgery scheduling problem is similar to the parallel machine scheduling problem. The ABC algorithm has been applied to several parallel machine scheduling problems [17,18,19,20], and the results are promising. Hence, we chose the ABC algorithm to solve the studied problem. The ABC algorithm works in an iterative process. The colony of artificial bees consists of three groups: employed bees, onlooker bees, and scout bees. Each employed bee corresponds to a food source. The location of a food source corresponds to a possible solution to the studied problem and the nectar amount of the food source represents the quality of the solution. Employed bees are responsible for performing simple and quick exploitation (neighborhood search) on available food sources and for informing the onlooker bees about the nectar amounts of the sources. Next, the onlooker bees select existing food sources and conduct more detailed exploitation based on the information they receive from the employed bees. The higher the nectar amount of a food source, the higher the probability it would be selected by onlooker bees. If the quality (fitness) of the food source (solution) cannot be improved after performing simple and quick exploitation, the corresponding food source is abandoned and the employed bee is converted to a scout bee. The scout bee will then seek a new food source randomly. We describe the implementation of the ABC algorithm as follows.

### 4.1. Coding Scheme

We adopt the same coding scheme as used in [2] to represent a solution on hand. The surgery symbols are denoted using integers. The partition symbols denoted with “R” or “D” designate the partition of surgeries to operating rooms and operating days, respectively. Since not all the surgeries can be scheduled into the current planning horizon, the length of a solution varies. For example, suppose 10 surgeries are waiting to be scheduled into two days, each day has two operating rooms available. A solution can be represented as follows: (9 2 R 1 D 7 10 R 3 6 4). The corresponding schedule is surgeries 9 and 2 in operating room 1 on day 1; surgery 1 in operating room 2 on day 1; surgeries 7 and 10 in operating room 1 on day 2; and surgeries 3, 6, and 4 in operating room 2 on day 2. Surgeries 5 and 8 are not assigned. Another possible solution (8 3 5 R D 7 2 R 9 6) represents surgeries 8, 3, and 5 in operating room 1 on day 1; no surgery in operating room 2 on day 1; surgeries 7 and 2 in operating room 1 on day 2; and surgeries 9 and 6 in operating room 2 on day 2. Surgeries 1, 4, and 10 are not assigned.

### 4.2. Initialization

In the ABC algorithm, each employed bee is dispatched to work on a specific food source and each food source is associated with a solution. In order to give the ABC algorithm good initial solutions, we inseredt two solutions generated by using the MEDD and MLPT heuristics into the initial solutions. The remaining initial solutions were randomly generated by using the following RandomlyGenerateSolutionScheme to make up *SN* initial population.

#### Randomly Generate Solution Scheme

Step 1. For a given problem instance, sort all surgeries by the EDD rule. Break any ties by using the LPT rule. Save all the sorted surgeries in set U. Initially, set $\pi \;=\;\left\{\mathrm{null}\right\}$.

Step 2. If $U\;=\;\left\{\mathrm{null}\right\}$, go to step 3. Otherwise, try to schedule the first surgery x,x∈U into schedule π. If $D_{x}\leq H$, randomly choose one operating room *k* on day $d,\;d\leq D_{x}$ and add surgery *x* into operating room *k* on day *d*, update schedule π, and remove surgery *x* from U. If $D_{x}>H$, randomly choose one operating room *k* on day d, d≤H and add surgery *x* in operating room *k* on day *d*, update schedule π, and remove surgery *x* from U. Go back to step 2.

Step 3. Apply RecoveryScheme (described in Section 4.3) to generate a feasible solution π.

### 4.3. RecoveryScheme

The studied operating room scheduling problem is a highly constrained combinatorial optimization problem. A feasible solution needs to meet all the due dates of scheduled surgeries, to meet each surgeon’s maximum available operating time on each day, and to meet the maximum permissible overtime of each operating room on each day. All those constraints are hard constraints. Violations of any of those hard constraints will lead to infeasible solutions. As the number of surgery increases, the number of hard constraints increases, and the probability of generating infeasible solutions becomes very high. Hence, we proposed a RecoveryScheme that can recover an infeasible solution into a feasible solution. We describe the RecoveryScheme as below. For a given schedule π, assuming *B* and $B^{c}$ are the set of scheduled surgeries and the set of unscheduled surgeries (i.e., $B^{c}$ is $B^{\prime }$s complementary set where $B\;+\;B^{c}\;=\;\Omega )$. In step 1, the RecoveryScheme removes surgeries that violate due date constraints to $B^{c}$. In step 2, it removes surgeries that violate the constraint $f_{k}^{d}$($\pi )\leq RT_{k}^{d}\;+\;OT_{k}^{d}$ from B to $B^{c}$. In step 3, it removes surgeries that violate the constraint $g_{l}^{d}\left(\pi \right)\leq A_{l}^{d}$ from B to $B^{c}$. In step 4, it sorts all surgeries in set $B^{c}$ by the EDD rule. For surgeries *x*, where $D_{x}\leq H$, it first tries to find an operating room *k* on day *d* that adds surgeries *x* into operating room *k* on day *d* that can keep schedule π feasible. If *k* and *d* are found, then it adds surgery *x* into operating room *k* on day *d*, removes surgery *x* from $B^{c}$, updates schedule π, and goes back to step 4. If *k* and *d* are not found, it randomly chooses one operating room *k* on day $d,\;d\leq D_{x}$, adds surgery x to operating room *k* on day d, removes surgery *x* from $B^{c}$, and updates schedule π. Here, the schedule π might violate the constraints $f_{k}^{d}$($\pi )\leq RT_{k}^{d}\;+\;OT_{k}^{d}$ or $g_{l}^{d}\left(\pi \right)\leq A_{l}^{d}$ again. Step 4 is repeated until there are no more surgeries $x,\;x\in B^{c}$ that have due dates smaller or equal to the planning horizon. For the remaining surgeries in set $B^{c}$, step 5 tries to schedule more surgeries $x,x\in B^{c}$ randomly to operating room *k* on day d if it satisfies $f_{k}^{d}$($\pi )\leq RT_{k}^{d}\;+\;OT_{k}^{d}$ and $g_{l}^{d}\left(\pi \right)\leq A_{l}^{d}$. Step 6: if schedule π is feasible, then RecoveryScheme outputs the final feasible schedule π and terminates the recovery process. Otherwise, it goes back to step 1 and continues the recovery process.

RecoveryScheme

Step 1: For a given schedule π, update *B* and $B^{c}$. Remove surgeries x,x∈B that are scheduled later than their due dates ($d_{i}>D_{i}$) to $B^{c}$. Update schedule π.

Step 2: Calculate $f_{k}^{d}$(π) ∀d,k. Find $k^{*}$ and $d^{*}$ that satisfies $f_{k^{*}}^{d^{*}}$(π) > $RT_{k^{*}}^{d^{*}}\;+\;OT_{k^{*}}^{d^{*}}\;\forall d,k$. Randomly remove surgery (surgeries) *x* that is (are) scheduled in operating room $k^{*}$ on date $d^{*}$ from *B* to $B^{c}$ until $f_{k^{*}}^{d^{*}}$($\pi )\leq RT_{k^{*}}^{d^{*}}\;+\;OT_{k^{*}}^{d^{*}}$. Update schedule π.

Step 3: Calculate $g_{l}^{d}\left(\pi \right)$ ∀d,l. Find $l^{*}$ and $d^{*}$ that satisfy $g_{l^{*}}^{d^{*}}\left(\pi \right)>A_{l^{*}}^{d^{*}}\;\forall d,k$. Randomly remove surgery (surgeries) *x*, $x\in \Omega_{l^{*}}$ that is (are) scheduled on $d^{*}$ from *B* to $B^{c}$ until $g_{l^{*}}^{d^{*}}\left(\pi \right)\leq A_{l^{*}}^{d^{*}}$. Update schedule π.

Step 4: Sort the surgeries into set $B^{c}$ by the EDD rule. Try to schedule surgeries $x,x\in B^{c}$, $x\in \Omega_{l}$, $D_{x}\leq H$ into operating room *k* on day *d* if it satisfies $f_{k}^{d}$($\pi )\leq RT_{k}^{d}\;+\;OT_{k}^{d}$ and $g_{l}^{d}\left(\pi \right)\leq A_{l}^{d}$. Try *k* = 1 first, if *k* is not feasible, then try *k* = *k* + 1 until k > $M_{d}$. Similarly, try *d* = 1 first; if *d* = 1 is not feasible, then try *d* = *d* + 1 until *d >* $D_{x}$. If *k* and *d* are found, then add surgery *x* into operating room *k* on day *d*, remove surgery *x* from $B^{c}$ to *B*, update schedule π, and go back to step 4. Otherwise, randomly choose one operating room *k* on day $d,\;d\leq D_{x}$. Add surgery x into operating room *k* on day *d*, remove surgery *x* from $B^{c}$ to *B*, and update schedule π. Repeat step 4 until there are no more surgeries $x,\;x\in B^{c}$ that satisfies $D_{x}\leq H$.

Step 5: For the remaining surgeries in set $B^{c}$. Let $U\;=\;B^{c}$. Choose the first surgery x,x∈U. Randomly choose one operating room *k* on day *d* from schedule π. If adding surgery x into operating room *k* on day *d* satisfies $f_{k}^{d}$($\pi )\leq RT_{k}^{d}\;+\;OT_{k}^{d}$ and $g_{l}^{d}\left(\pi \right)\leq A_{l}^{d}$, then insert it into and update schedule π. Otherwise, remove surgery x from U. Repeat step 5 until $U\;=\;\left\{null\right\}$.

Step 6: If schedule π is feasible, then output the final feasible schedule π and terminate the recovery process. Otherwise, go back to step 1.

### 4.4. Local Search Schemes

Two local search schemes were proposed in this research. They are used to intensify and diversify the solution pool. The proposed local search schemes are described below.

#### 4.4.1. Internal Swap

Step 1: For a given schedule π, randomly choose two surgeries $x,y\;\left(x,y\in B\right)$

Step 2: If $D_{x}\geq d_{y}$, $D_{y}\geq d_{x}$, and x,y are not scheduled in the same operating room on the same date, then swap x and y and update schedule π. Otherwise, go back to step 1.

Step 3: Apply RecoveryScheme to schedule π.

The InternalSwap tries to find two scheduled surgeries *x* and *y* from a given schedule π. Step 2 ensures that the swapping of surgeries *x* and *y* satisfies $d_{i}\leq D_{i}$ constraints. Step 3 ensures that the schedule π is feasible after swapping.

#### 4.4.2. External Swap

Step 1: For a given schedule π, randomly choose one surgery x, x∈B and one unscheduled surgery $y,\;y\in B^{c}$.

Step 2: If $D_{x}>H$, then swap x and y and update schedule π. Otherwise, go back to step 1.

Step 3: Apply RecoveryScheme to schedule π.

The ExternalSwap tries to find a scheduled surgery *x* and an unscheduled surgery *y* from a given schedule π. Step 2 ensures that the swapping of surgeries *x* and *y* satisfies the constraints $d_{x}\leq D_{x}$ and $d_{y}\leq D_{y}$. Step 3 ensures that schedule π is feasible after swapping.

#### 4.4.3. InternalInsertion

Step 1: For a given schedule π, randomly choose one surgery x, x∈B. Suppose that surgery x is scheduled in operating room $k^{\prime }$ on day $d^{\prime }$. Randomly choose one operating room *k* ($k\neq k^{\prime }$) on day *d* ($d\neq d^{\prime }$).

Step 2: If $D_{x}\geq d$, then insert surgery x into operating room *k* on day *d* and update schedule π. Otherwise, go back to step 1.

Step 3: Apply RecoveryScheme to schedule π.

InternalInsertion tries to find a scheduled surgery *x* and one operating room *k* on day *d* from a given schedule π. Step 2 ensures that surgery *x* inserted into operating room *k* on day *d* satisfies the constraint $d_{x}\leq D_{x}$. Step 3 ensures that the schedule π is feasible after insertion.

#### 4.4.4. ExternalInsertion

Step 1: For a given schedule π, randomly choose one surgery $x,\;x\in B^{c}$ and randomly choose one operating room *k* on day *d*.

Step 2: If $D_{x}\geq d$, then insert surgery x into operating room *k* on day *d* and update schedule π. Otherwise, go back to step 1.

Step 3: Apply RecoveryScheme to schedule π.

ExternalInsertion tries to find an unscheduled surgery *x* and one operating room *k* on day *d* from a given schedule π. Step 2 makes sure that surgery *x* inserted into operating room *k* on day *d* satisfies the constraint $d_{x}\leq D_{x}$. Step 3 ensures that the schedule π is feasible after insertion.

Our initial trials indicated that ExternalInsertion outperformed ExternalSwap, that ExternalSwap outperformed InternalInsertion, and that InternalInsertion outperformed InternalSwap. In our ABC, we combined and used different local search schemes to form two types of local search strategies, namely ExplorationProcess and ExploitationProcess. The ExplorationProcess is a simple and efficient way to diversify the current solution pool; the ExploitationProcess is a greedy search to intensify a given solution. ExplorationProcess and ExploitationProcess are described below.

#### 4.4.5. ExplorationProcess

Step 0. Initially, set s = 0.

Step 1. Randomly generate an integer number *aa* from a uniform distribution (1,4). If *aa* = 1, go to step 2. If *aa* = 2, go to step 3. If *aa* = 3, go to step 4. If *aa* = 4, go to step 5.

Step 2. Perform InternalSwap on schedule π. If a smaller cost is found, then update schedule π and terminate the procedure. Otherwise, *s* = *s* + 1. If *s* < *S*, then go back to step 1. Otherwise, go to step 6.

Step 3. Perform InternalInsertion on schedule π. If a smaller cost is found, then update schedule π and terminate the procedure. Otherwise, *s* = *s* + 1. If *s* < *S*, then go back to step 1. Otherwise, go to step 6.

Step 4. Perform ExternalSwap on schedule π. If a smaller cost is found, then update schedule π and terminate the procedure. Otherwise, *s* = *s* + 1. If *s* < *S*, then go back to step 1. Otherwise, go to step 6.

Step 5. Perform ExternalInsertion on schedule 

π. If a smaller cost is found, then update schedule π and terminate the procedure. Otherwise, *s* = *s* + 1. If *s* < *S*, then go back to step 1. Otherwise, go to step 6.

Step 6. Randomly generate an integer number *bb* from a uniform distribution (1,2). If *bb* = 1, then output the final schedule π; otherwise, discard schedule π and apply RandomlyGenerateSolutionScheme to generate a new schedule.

#### 4.4.6. ExploitationProcess

Step 1. Apply ExternalInsertion. If a smaller cost is found, then go to step 1; otherwise, go to step 2.

Step 2. Apply ExternalSwap. If a smaller cost is found, then go to step 2; otherwise, go to step 3.

Step 3. Apply InternalInsertion. If a smaller cost is found, then go to step 3; otherwise, go to step 4.

Step 4. Apply InternalSwap. If a smaller cost is found, then go to step 4; otherwise, go to step 5.

Step 5. Terminate ExploitationProcess.

### 4.5. Fitness Value and Selection

We defined the objective value of schedule π as the total operating cost ($\sum_{d\;=\;1}^{H}\sum_{k\;=\;1}^{M_{d}}C_{k}^{d}$). Since we are minimizing the objective, the fitness value of a solution π is inversely proportional to its objective (π), as shown in Formula (11). Next, we applied a basic roulette wheel [21] selection mechanism to the sampling space ($p_{s})$ to form a new population. A roulette wheel selects a new population based on a fitness-proportional probability distribution. A bee’s probability for selection can be calculated from Formula (12).

(11) $$f\left(h\right)\;=\;\frac{1}{objective\left(\pi \right)}\;=\;\frac{1}{\sum_{d\;=\;1}^{H}\sum_{k\;=\;1}^{M_{d}}C_{k}^{d}}$$

(12) $$prob_{h}\;=\;\frac{f\left(h\right)}{\sum_{s\;=\;1}^{p_{s}}f\left(s\right)},h\;=\;1,\ldots ,\;p_{s}$$

### 4.6. Elitism Strategy

In each iteration, we saved the top MaxElite unique solutions and their attributes in the elite list (EliteSolutions). The elite solutions are sorted by nondecreasing order of total cost. When the current best solution does not update for several iterations, the search might get trapped into a local optimal. When this occurs, we apply an elitism strategy to help the search escape from the local optimal. Our elitism strategy is described below.

#### Elitism Strategy

Step 1: Apply ExternalInsertion ∀d,k. For a given schedule π, try to perform an external insertion for every surgery $x,\;x\in B^{c}$ to all operating rooms on all dates. Precisely, select the *r*th (*r* = 1,...,$\left|B^{c}\right|$) surgery *x* and try to insert it into operating room $k,\;k\;=\;1,\ldots ,M_{d}$ on day d, d = 1, …, H. If insert surgery x into operating room *k* on day *d* can keep the schedule feasible with a smaller cost than the cost before insertion, then insert it into and update schedule π, and update EliteSolutions. The procedure is repeated for the current schedule π until all surgeries in set $B^{c}$ have been examined.

Step 2: Apply ExternalSwapforAllPairs. For a given schedule π, try to perform an external swap for each pair of surgeries x, x∈B and $y,\;y\in B^{c}$. Specifically, select the *r*th (*r* = 1,..., $\left|B\right|$) surgery from the schedule in π and try to swap it with all surgeries in set $B^{c}$. If swapping x and y can keep the schedule feasible with a smaller cost than the cost before the swap, then swap the surgeries, update schedule π, and update EliteSolutions. The procedure is repeated for the current schedule π until all pairs of surgeries in set *B* and $B^{c}$ have been examined.

Step 3: Apply InternalInsertion ∀d,k. For a given schedule π, try to perform an internal insertion for every surgery x, x∈B to all feasible operating rooms on all feasible dates. Precisely, select the *r*th (*r* = 1,...,$\left|B\right|$) surgery x from schedule π. Assuming surgery x is scheduled in operating room $k^{\prime }$ on day $d^{\prime }$. Try to insert surgery x into operating room $k,\;k\;=\;1,\ldots ,M_{d}$, $k\neq k^{\prime }$ on day $d,\;d\;=\;1,\;\ldots ,\;D_{x}$, $d\neq d^{\prime }$. If inserting surgery x into operating room *k* on day *d* can keep the schedule feasible with a smaller cost than the cost before insertion, then insert it into and update schedule π, and update *EliteSolutions*. The procedure is repeated for the current schedule π until all surgeries in set *B* have been examined.

Step 4: Apply InternalSwapforAllPairs. For a given schedule π, try to perform an internal swap for each pair of two surgeries $x,y\;\left(x,y\in B\right)$. specifically, select the *r*th (*r* = 1,..., $\left|B\right|$) surgery x from the schedule π. Assuming that surgery x is scheduled in the operating room $k^{\prime }$ on day $d^{\prime }$, Try to swap it with all other surgeries in set *B* but exclude surgeries that are also scheduled in operating room $k^{\prime }$ on day $d^{\prime }$. If swapping x and y can keep the schedule feasible with a smaller cost than the cost before the swap, then swap the surgeries, update schedule π, and update EliteSolutions. The procedure is repeated for the current schedule π until all pairs of two surgeries in set *B* have been examined.

### 4.7. The Implementation of the Proposed ABC Algorithm

**Figure 3:**
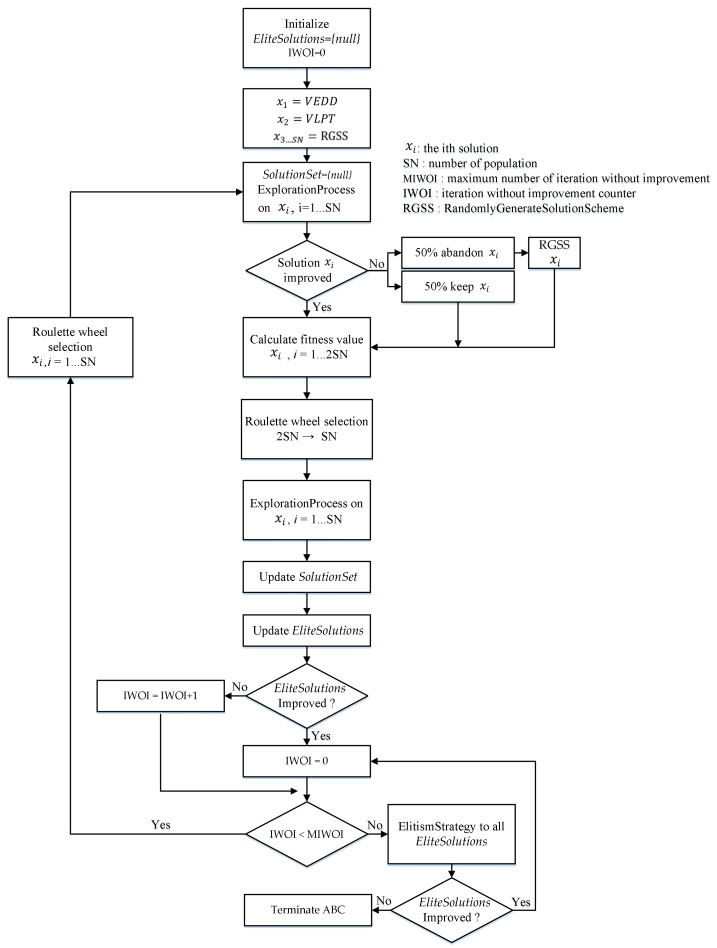
The flow chart of the artificial bee colony (ABC) algorithm.

Based on the details (coding, initialization, a recovery scheme, local search schemes, fitness value and selection, and elitism strategy) presented in the previous subsection, the implementation of the proposed ABC algorithm is described as follows. We also provide a flow chart of the proposed ABC in Figure 3.

Step 0: Let *SolutionSet* be the set of solutions found by all bees and EliteSolutions be the set of elite solutions. Initially, set EliteSolutions = {null}, and iteration without improvement counter IWOI = 0.

Step 1: Insert the two solutions generated by using the MEDD and MLPT heuristics. The remaining initial solutions are randomly generated by using RandomlyGenerateSolutionScheme to make up the *SN* initial population. Each initial solution corresponds to an employed bee that is associated with a food source.

Step 2: Set SolutionSet = {null}. Each employed bee works on a food source (a solution) and evaluates their nectar amounts by imposing ExplorationProcess to see if a better solution can be found. Once a better solution π has been found, the employed bee will save the new food source, update π, and terminate the initial exploration. If the quality of the food source (a solution) cannot be improved after performing a maximum allowable number of iterations (*S*), there is a 50% probability that the employed bee will keep the food source. In the other 50% probability, the food source is abandoned. If the food source is abandoned, the employed bee is converted to a scout bee. The scout bee will then start to search for a new food source by using RandomlyGenerateSolutionScheme to randomly generate a new solution. After all the employed bees (*SN*) have finished with the exploration process, the ABC algorithm sorts all the solutions in nondecreasing order of total cost.

Step 3: After all employed bees have finished the exploration process, the onlooker bees will receive nectar information of the food sources from the employed bees. The *SN* onlookers then select existing food sources (the enlarge food source size is 2×SN) to be further explored in a probabilistic manner by using Formula (12). The better the solution, the higher the probability it would be selected by the onlooker bees.

Step 4: After the onlooker bee has selected a food source, it conducts a more detailed neighborhood search on it by applying ExploitationProcess. After all onlooker bees (*SN*) have finished the exploitation process, the ABC algorithm sorts all the solutions in nondecreasing order of total cost and saves them into SolutionSet.

Step 5: Check whether any solutions in SolutionSet has outperformed the solutions in EliteSolutions. If yes, update EliteSolutions and set IWOI = 0; otherwise, set IWOI = IWOI + 1.

Step 6: Check whether IWOI has reached the maximum number of non-improving iterations required to trigger the search procedure for elite solutions (IWOI *>* MIWOI): if yes, apply the ElitismStrategy to all solutions stored in EliteSolutions. Otherwise, select SN employed bees from SolutionSet by using Formula (12) and go back to step 2.

Step 7: Check whether the EliteSolutions has been updated. If yes, set IWOI = 0 and select SN employed bees from SolutionSet by using Formula (12) and go back to Step 2. Otherwise, go to step 8.

Step 8: Terminate the ABC algorithm and report the final best solution in EliteSolutions.

We conducted some preliminary experiments to check the performance of the ABC algorithm for different parameter settings. In order to find a tradeoff between solution quality and computation time, the following settings are used for the parameters in the ABC algorithm.

*SN* = 250

*S* = 150

MaxElite = 5

MIWOI = 20

## 5. Computational Results

In this section, we present several computational results regarding the performance of the proposed MEDD heuristic, MLPT heuristic, and ABC algorithm. The problems were solved in the following environment:

Hardware for running the algorithm: ASUS TeK (AMD Ryzen 7 4700 U 2.00 GHz, memory: 8 GB).

Algorithm development environment: Microsoft Visual C + + 2019.

Software for running the MIP model: AMPL with CPLEX 11.2 solver.

The testing data were generated as follows:

The planning horizon is 5 days (a week). According to Combes et al. [22] and Fei et al. [3], the Pearson III distribution fits the distribution of surgery’s operating time very well. Hence, the operating times of surgeries are generated from the Pearson III distribution. In this study, test problems were generated in a manner similar to that of [3].

The surgery operating time, $t_{i}$, was generated from the Pearson III distribution in the range (40, 150) with the mean at 90 and a standard deviation of 15 (unit: minute).

The due date of surgery *i*, $D_{i}$, was generated from a uniform distribution in the range (1,14) (unit: day).

Each surgery was randomly assigned to a surgeon in advance. For each surgery *i*, we randomly generated an integer number *l* from a uniform distribution (1,8). Then, surgery *i* was assigned to surgeon *l*.

The cost ratio of ordinary working hours and overtime ones was set to α = 1.5.

The number of surgeons was set to 8, and the maximum operating time (unit: hour) available of surgeons during the planning horizon was as follows [3].

From Monday to Friday ($A_{l}^{d}$),

Surgeon 1: (8,0,7,0,6)

Surgeon 2: (8,4,5,6,5)

Surgeon 3: (12,3,6,7,8)

Surgeon 4: (5,3,4,8,10)

Surgeon 5: (6,5,0,6,8)

Surgeon 6: (6,0,5,7,9)

Surgeon 7: (0,6,6,6,9)

Surgeon 8: (0,6,6,8,10)

The number of operating rooms considered was set to 6, and the regular opening hours and maximum overtime hours during the planning horizon were given as follows [3].

Regular opening hours from Monday to Friday ($RT_{k}^{d}$):

Operating room 1: (8,5,4,6,5)

Operating room 2: (5,7,8,5,4)

Operating room 3: (7,6,7,8,6)

Operating room 4: (4,5,8,4,8)

Operating room 5: (8,8,5,7,4)

Operating room 6: (8,-,5,4,7), where “-“ means the operating room is not available.

Maximum overtime hours from Monday to Friday ($OT_{k}^{d}$. ):

Operating room 1: (2,3,3,0,2)

Operating room 2: (0,0,1,2,2)

Operating room 3: (2,2,2,2,1)

Operating room 4: (1,3,1,2,0)

Operating room 5: (2,0,2,2,0)

Operating room 6: (0,-,2,2,0), where “-“ means the operating room is not available.

The number of surgeries waiting to be operated Ω was set to 40, 50, 60, …, 150. We used Ω = 40–110 to represent small problems and Ω = 120–150 to represent large problems. For each setting of Ω, 20 instances were randomly generated. In total, there are 240 instances that are generated and tested.

For small problems, we compared the MEDD heuristic, MLPT heuristic, and ABC algorithm with the optimal solutions found using the MIP model. Table 2 shows the results of small problem instances. For each tested surgery size Ω, we report the average of 20 randomly generated instances. The first column reports the optimal solution obtained by solving the MIP model. Table 2 shows that the MEDD heuristic outperforms the MLPT and that the ABC algorithm improved the initial solutions obtained by the two heuristics significantly. The MEDD heuristic is 8.47% deviated from the optimal solution on average, the MLPT is 17.68% deviated from the optimal solution on average, and the ABC algorithm is 0.1% deviated from the optimal solution on average. The proposed ABC algorithm can find optimal solutions for all of the tested instances of surgery size Ω = 40, 50, 60, 70, 80, 90, and 100.

For a surgery size larger than 110, the problems fail to be solved by the MIP model due to an out of memory error. Hence, for large problem instances, we compared the MEDD heuristic and MLPT heuristic with the ABC algorithm. Table 3 shows that the MEDD heuristic outperforms the MLPT and that the ABC algorithm has improved the initial solutions obtained by the two heuristics significantly. The cost obtained by the ABC algorithm decreases dramatically when compared to the cost obtained by the two heuristics. In all, the MEDD heuristic is 471.8% deviated from the ABC algorithm on average and the MLPT is 669.72% deviated from the ABC algorithm.

**Figure 4:**
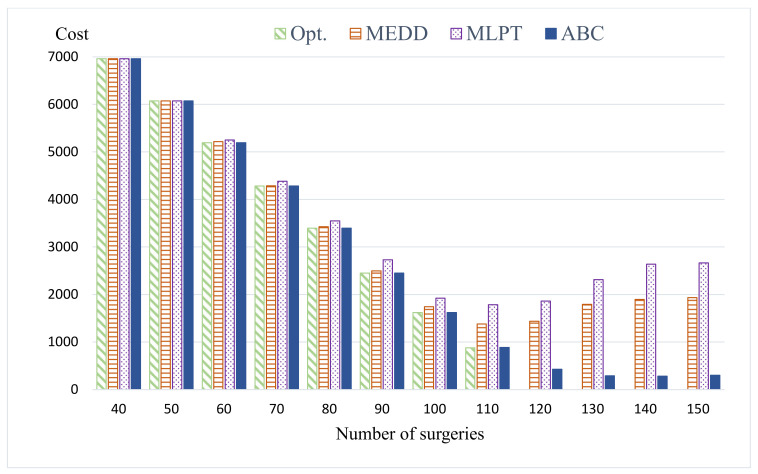
Comparison of four methods.

Moreover, we took the costs from Table 2 and Table 3 and demonstrated the comparison of four methods in Figure 4. For the number of surgeries between 40 and 100, the ABC algorithm was able to find the cost that is the same as the optimal solution for all testing instances. For the number of surgeries between 110 and 150, the mathematical model cannot find an optimal solution as it ran out of memory. Hence, Figure 4 does not report the results of optimal solutions for the number of surgeries between 110 and 150. For the number of surgeries between 110 and 150, the ABC algorithm outperforms the MEDD and MLPT in terms of total cost significantly.

Table 4 shows the computation time of the MIP model, heuristics, and the ABC algorithm. The MIP model spent more time finding an optimal solution than the heuristics and the ABC algorithm. The MIP model spent about 446.31 s to find an optimal solution for 100 surgery problem instances on average. As mentioned before, the MIP model can only solve small problem instances. Two heuristics can find a feasible solution in less than a second for all problem instances. As the number of surgery increases, the computation time of the ABC algorithm increases. On average, the ABC spent about 98.38 s to find a feasible solution that is significantly better than the two heuristics.

The result is the average of 20 randomly generated problem instances.

We also report the number of scheduled surgeries for each surgery size *Ω*. The result shows that almost all surgeries have been scheduled into the planning horizon for *Ω* between 40 and 100 problem instances. Table 4 shows that the total cost of all methods is high for Ω = 40–100 problem instances. This is probably because the operating rooms are underused and unused costs occur. For Ω between 120 and 150, the ABC algorithm schedules about 115 surgeries into the planning horizon on average, and the total costs are relatively low compared to other problem sizes. This is probably because the capacities of the operating rooms are full. In another word, the operating rooms are fully used. The ABC algorithm tried to schedule surgeries into the regular opening time and only a small amount of overtime is needed.

**Figure 5:**
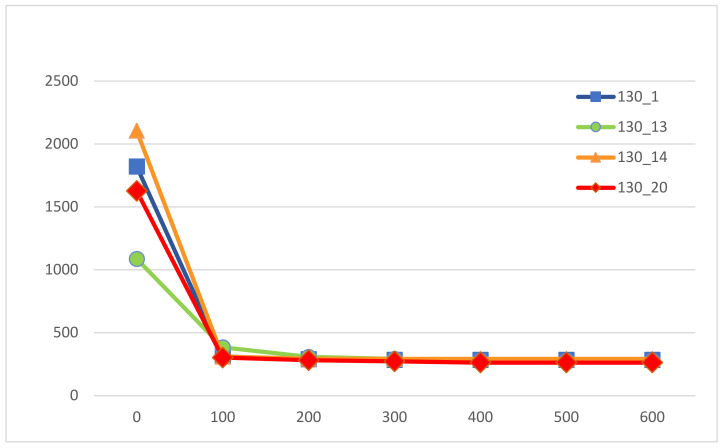
The converge curves of the proposed ABC.

Furthermore, we examined the convergence curves of the proposed ABC. We maintain the termination condition of the ABC but count the number of iterations until the ABC is terminated. Figure 5 shows four typical convergence curves for some of the test instances of Ω = 130. Specifically, instances 1, 13, 14, and 20 are chosen and reported in Figure 5. The other instances have similar results. From the curves, the ABC has a steep descent for early generations. The ABC, on average, takes 400 iterations to converge to the best solution for the 20 tested instances of surgery size Ω = 130.

Since this research studied the same problem as [3] and we used the same manner as [3] to generate testing data, we made a comparison of our results with [3]. In [3], the authors first constructed a mathematical model that assigned surgeries into operating rooms within one week by using the open scheduling strategy. Next, they reformulated the mathematical model as a set-partitioning model and then solved it by using a column-generation based heuristic (CGBH) procedure with four criteria, namely H1, H2, H3, and H4. The proposed CGBH procedure with four criteria was compared with each other to find a solution with the best performance. Moreover, the best approximate solution, obtained by the CGBH procedure after running all the criteria proposed, was compared with the lower bound obtained by an explicit column generation (CG) procedure (the optimal solution of the linear relaxation of the set-partitioning model). They tested surgery size Ω = 40, 60, 80, 100, 120, 140, and 160. For each surgery size Ω, 10 problem instances were generated and tested. Therefore, totally, 70 problem instances are generated and tested. Instead of solving a mathematical model to obtain an optimal solution, the work in [3] solves the explicit CG procedure to obtain a lower bound. All the tested instances of surgery size Ω = 40, 60, and 80 are optimally solved by the explicit CG procedure. As for those instances of large size Ω = 100, 120, 140, and 160, the CGBH procedure with criterion H2 can obtain the best solution among those four criteria in most of the cases once it obtains a feasible solution. However, H2 cannot always obtain a feasible solution, especially in the scenario of Ω = 160. In fact, the CGBH procedure with four criteria cannot always obtain a feasible solution for each instance. Among the 70 tested instances, 100% of them can be solved by the CGBH procedure with criterion H1 within 10 times of execution; 74.29% can be solved by the CGBH procedure with criterion H2; 72.85% can be solved by the CGBH procedure with criterion H3; and 71.43% can be solved by the CGBH procedure with criterion H4. Next, they compared the approximate solution obtained by H with the lower bound obtained by the explicit CG procedure. H represents the objective value of the best approximate solution, selected among approximate solutions obtained by the CGBH procedure after running all those four criteria. They reported that 45 instances (45/70 = 64.29%) can be optimally solved by the explicit CG procedure. For surgery size Ω = 100, 120, 140, and 160, H deviated by 0.13%, 2.2%, 4.73%, and 34.58% from the lower bound on average ((H-lower bound)/H). Lastly, the work in [3] showed the computational time used by the CGBH procedure with four criteria. The largest one, surgery size Ω = 140, took about 15 min by H.

In this research, all the tested instances of surgery sizes Ω = 40, 50, 60, 70, 80, 90, 100, and 110 are optimally solved by the modified MIP model. The proposed ABC algorithm can find optimal solutions for all the tested instances of surgery size Ω = 40, 50, 60, 70, 80, 90, and 100. The MIP model cannot solve problems of surgery size larger than 110 due to memory issues. Hence, for large problem instances, we compared the MEDD and MLPT heuristics with the ABC algorithm. The results indicate that the ABC algorithm improved the initial solutions obtained by the two heuristics significantly. The cost found by the ABC algorithm decreases dramatically when compared to the cost found by the two heuristics. In all, the MEDD heuristic deviated by 471.8% from the ABC algorithm on average and the MLPT deviated by 669.72% from the ABC algorithm. Moreover, the ABC algorithm and the two proposed heuristics can always obtain a feasible solution for all the tested problem instances. Lastly, Table 4 shows the computational time of the four methods. The largest one, surgery size Ω = 150, takes about 6.7 min using the ABC algorithm. Hence, we can conclude that the ABC algorithm can find better solutions more efficiently than the CGBH procedure with the four criteria proposed in [3].

## 6. Conclusions and Future Work

An operating room department is a unit with the high cost and high revenue. Hence, it is important to ensure the efficient use of operating rooms. In this research, we have considered constructing a weekly surgery schedule with an open scheduling strategy by taking the availabilities of surgeons and operating rooms into account. The objective is to minimize total operating costs. We first provided a revised mathematical model that can find optimal solutions for small problem instances. Moreover, two modified heuristics were proposed that can find feasible solutions for the studied problem very quickly. Finally, an ABC algorithm that incorporates initial solutions, a recovery scheme, local search schemes, and elite strategy was proposed. The computational results show that, for surgery size Ω = 40–100, the ABC algorithm found optimal solutions for all the tested problems. For surgery size Ω = 120–150, the ABC algorithm improved the initial solutions found by the two proposed heuristics significantly. The solutions of MEDD heuristic deviated from the solutions of ABC algorithm by 471.8% on average, and for the results of MLPT, the deviation was 669.72%. We concluded that the ABC algorithm can solve the studied problems efficiently with less operating cost and higher utilization of operating rooms.

Since all test data were generated randomly, our future work can involve collaborating with local hospitals and testing our algorithm on real data to make the results of our research more practical. Further work can also include studying more complex problems with more constraints and variables, such as the availability of anesthetists, nurses, and recovery beds.

## Figures and Tables

**Table 1:** Notations used in this article.

*Ω*	number of surgeries waiting to be operated
$M_{d}$	number of operating rooms available in a hospital on day *d*
*H*	number of days for planning horizon (normally one week, *H* = 5)
*L*	number of surgeons
$\Omega_{l}$	set of surgeries that are assigned to surgeon *l*
$A_{l}^{d}$	maximum operating time available of surgeon *l* on day *d*
$t_{i}$	operating duration of surgery *i*
$D_{i}$	deadline of surgery *i* in days (the day before which surgery *i* must be performed)
$RT_{k}^{d}$	regular opening time of operating room *k* on day *d*
$OT_{k}^{d}$	maximum permissible overtime of operating room *k* on day *d*
$d_{i}$	the scheduled operating day of surgery *i* in a given schedule (π)
$f_{k}^{d}$	the sum of operating times of surgeries that have already been scheduled in operating room *k* on day *d*, $f_{k}^{d}\;=\;\sum_{i\in \Omega }t_{i}x_{ik}^{d}\;\forall k,d$
$g_{l}^{d}$	the sum of operating times of surgeries that are assigned to surgeon *l* on day *d*, $g_{l}^{d}\;=\;\sum_{k\;=\;1}^{M_{d}}\sum_{i\in \Omega_{l}}^{}t_{i}x_{ik}^{d}$ ∀d, l
$C_{k}^{d}$	operating cost of operating room *k* on day *d*, $C_{k}^{d}\;=\;UC_{k}^{d}\;+\;\alpha OC_{k}^{d}$
α	cost ratio of ordinary working hours and overtime ones
*SN*	number of population
*S*	the maximum number of exploration process for an employed bee
*MaxElite*	the maximum number of elite solutions that can be stored
*MIWOI*	maximum number of iteration without improvement

**Table 2:** The performance of the heuristics and the ABC algorithm for small problem instances.

Number of Surgeries Ω	Cost				$\mathrm{GAP}\;=\;\frac{\left(Heuristic-Opt\right)}{Opt}\;\times \;100$		
	Opt.	MEDD	MLPT	ABC	MEDD	MLPT	ABC
40	6961.50	6961.50	6961.50	6961.50	0.00%	0.00%	0.00%
50	6073.20	6073.20	6073.20	6073.20	0.00%	0.00%	0.00%
60	5194.70	5214.85	5252.25	5194.70	0.34%	1.11%	0.00%
70	4280.95	4285.60	4383.40	4280.95	0.11%	2.39%	0.00%
80	3394.85	3423.50	3547.75	3394.85	0.88%	4.50%	0.00%
90	2450.95	2495.25	2728.55	2451.00	1.81%	11.33%	0.00%
100	1617.55	1740.33	1923.18	1617.55	7.59%	18.89%	0.00%
110	878.25	1378.83	1784.85	885.35	57.00%	103.23%	0.81%
Average	3856.49	3946.63	4081.83	3857.39	8.47%	17.68%	0.10%

Opt. is obtained by solving the mixed integer programming (MIP) model. The result is the average of 20 randomly generated instances.

**Table 3:** The performance of the heuristics and the ABC algorithm for large problem instances.

Number of Surgeries Ω	Cost			$\mathrm{GAP}\;=\;\frac{\left(Heuristic-ABC\right)}{ABC}\;\times \;100\%$	
	MEDD	MLPT	ABC	MEDD	MLPT
120	1436.28	1858.70	427.28	236.15%	335.01%
130	1790.35	2310.48	286.45	525.01%	706.59%
140	1894.03	2638.48	278.93	579.05%	845.94%
150	1934.23	2664.60	298.95	547.01%	791.32%
Average	1763.72	2368.06	322.90	471.80%	669.72%

**Table 4:** The computational time of the MIP model, heuristics, and the ABC algorithm.

Number of Surgeries Ω	Computation Time (s)				Number of Scheduled Surgeries			
	Opt.	MEDD	MLPT	ABC	Opt.	MEDD	MLPT	ABC
40	0.04	0.0012	0.0007	1.06	40	40	40	40
50	0.05	0.0013	0.0013	1.06	50	50	50	50
60	13.26	0.0014	0.0011	3.37	60	60	60	60
70	0.29	0.0015	0.0014	3.19	70	69.95	69.95	70
80	0.50	0.0025	0.0026	4.93	79.85	79.65	79.35	79.85
90	90.52	0.0024	0.0028	6.63	89.75	89.25	89.20	89.75
100	446.31	0.0029	0.0035	22.29	99.30	98.80	98.85	99.30
110	122.70	0.0017	0.0023	72.49	108.05	106.85	106.40	107.85
120	-	0.0031	0.0034	193.74	-	112.00	110.85	112.60
130	-	0.0040	0.0041	206.89	-	117.05	115.75	114.55
140	-	0.0021	0.0030	262.69	-	118.80	116.60	114.95
150	-	0.0024	0.0041	402.27	-	118.70	116.20	114.95
Average	-	0.0022	0.0025	98.38	-	88.42	87.76	87.82

## Data Availability

The data presented in this study are available on request from the corresponding author. The data are not publicly available due to privacy.
